# A Wideband Water-Based 3D-Printed Reflect–Transmit Antenna Array Toward mmWave Positioning Applications

**DOI:** 10.3390/s26041249

**Published:** 2026-02-14

**Authors:** Fahad Ahmed, Farooq Faisal, Noureddine Melouki, Peyman PourMohammadi, Hassan Naseri, Tarek Djerafi, Tayeb A. Denidni

**Affiliations:** Énergie Matériaux Télécommunications, Institut National de la Recherche Scientifique (INRS), Montreal, QC J3X 1P7, Canada; ahmed.fahad@inrs.ca (F.A.);

**Keywords:** bidirectional antennas, transmit–reflect array, millimeter-wave antennas, 3D-printed antennas, water-based dielectric, positioning and sensing

## Abstract

This paper presents a water-based reflect-transmit antenna (WBRTA) array for millimeter-wave (mm-wave) applications. The WBRTA array incorporates the low-permittivity polylactic acid (PLA)- and high-permittivity water-based unit cells. The low permittivity PLA unit cells provide better transmission, whereas the water-based unit cell offers good reflections due to a very high permittivity. Therefore, the WBRTA enables simultaneous beam splitting in reflection and transmission modes across a wider bandwidth. In addition, depending on the distribution and configuration of the water- and PLA-based unit cells, the WBRTA enables beam tilting of up to 45° in the reflection and transmission modes simultaneously. The proposed WBRTA offers peak gains of 25.2 dBi in transmission and 24 dBi in reflection at the central frequency. The corresponding sidelobe levels (SLLs) are −22 dB for transmission and −17 dB for reflection, while cross-polarization (x-pol) levels remain below −81 dB. In addition, the wide operational bandwidth, low sidelobe levels, and high polarization purity make the proposed WBRTA relevant as an enabling antenna structure for positioning-oriented sensing functions in future mmWave wireless systems.

## 1. Introduction

In recent years, reflect–transmit antennas (RTAs) have received significant attention over traditional antennas due to their compact size, high gain, and applications in communication systems. Bidirectional communication can reach users in multiple directions and can be implemented in systems that require simultaneous transmission and reception, making it suitable for space and ground communication [1,2,3,4]. In contrast, unidirectional antennas, such as simple reflectarrays (RAs) and transmitarrays (TAs), are not suitable for these environments due to their limitation to a single radiation direction [5,6,7,8,9,10,11,12]. Beyond communication, such bidirectional radiation characteristics are increasingly attractive for millimeter-wave (mmWave) positioning and sensing systems, where spatial coverage, angular diversity, and simultaneous illumination of multiple regions are required for accurate localization and target detection.

Beyond communication systems, the proposed reflect transmit antenna structure is particularly relevant for millimeter wave positioning and sensing applications. In such systems, wide angular coverage, low sidelobe levels, and high polarization purity are essential for accurate angle of arrival estimation and spatial discrimination of targets. The simultaneous beam splitting in reflection and transmission enables illumination of multiple spatial regions without mechanical steering, which is highly beneficial for indoor localization, device tracking, and environment sensing in future wireless networks. Owing to its wide bandwidth, stable phase behavior, and bidirectional radiation capability, the proposed WBRTA can serve as an efficient hardware platform for positioning oriented mmWave sensing systems.

In this perspective, various RTAs have been reported in the literature [13,14,15,16,17,18,19,20,21,22]. In Ref. [16], a multibeam RTA was proposed that provides beam splitting in both transmission and reflection modes. However, this design achieves combined functionality at the expense of requiring a two-array configuration. Likewise, a multiband bidirectional antenna array reported in [17] offers pattern reconfigurability in the frequency band of 7–13 GHz; however, the proposed design contains seven layers and pin diodes. In the literature, a bidirectional antenna has also been reported that offers low profile and high gain, but its performance is restricted to beam splitting only [18]. Another 3D-printed wideband RTA comprising dielectric material and three copper wires was reported in [19]. The proposed design can control the beam in both the reflection and transmission directions; however, this controllability is dependent on unequal dielectric thickness and the precise positioning of metallic vias. Recently, a bidirectional antenna employing a multilayer configuration with a partially reflective surface (PRS) was reported for dual-band operation; however, its radiation is confined to the boresight directions (0° and 180°) only, limiting its angular coverage and flexibility [20]. From a positioning and sensing perspective, these limitations, such as restricted angular coverage, narrow operational bandwidth, and high structural complexity, directly degrade angular resolution, spatial selectivity, and system scalability in practical mmWave sensing deployments.

All the aforementioned designs reported in the literature offer bidirectional functionality but suffer from drawbacks such as higher cross-polarization levels, higher sidelobe levels (SLLs), low aperture efficiency (AE), multilayer structures, active components such as diodes, bulky nonuniform three-dimensional structures with vias, or metal on both sides of the array. Consequently, their applicability is limited in many practical scenarios because of high fabrication costs, longer production times, overall bulkiness, and compromised performance in terms of aperture efficiency, gain, SLLs, and cross-polarization levels. Such constraints are particularly critical for wideband and multiband positioning and sensing systems, where low sidelobe levels and high polarization purity are essential for reliable angle-of-arrival estimation and localization accuracy.

This paper presents a simple, metalless, and cost-effective water-based reflect–transmit antenna (WBRTA) array for mmWave applications. The WBRTA splits beams in the reflection and transmission modes over a broad bandwidth and enables beam tilting, based on different configurations of unit cells, of up to 45° in the reflection and transmission modes simultaneously. Furthermore, the proposed 3D-printable WBRTA array is not only flat, with uniform thickness across the aperture, but also avoids the use of vias and metallic components. Owing to its flexible functionality, good aperture efficiency, low sidelobe levels, low cross-polarization, simple structure, and low fabrication cost, the proposed WBRTA exhibits greater potential than existing RTA arrays reported in the literature. These characteristics make the proposed array a promising hardware platform for wideband mmWave positioning-enabled wireless systems, where compactness, wide angular coverage, and stable wideband beamforming are important design enablers.

## 2. Unit Cell Design and Performance Evaluation

### 2.1. Unit Cell Design

**Figure 1:**
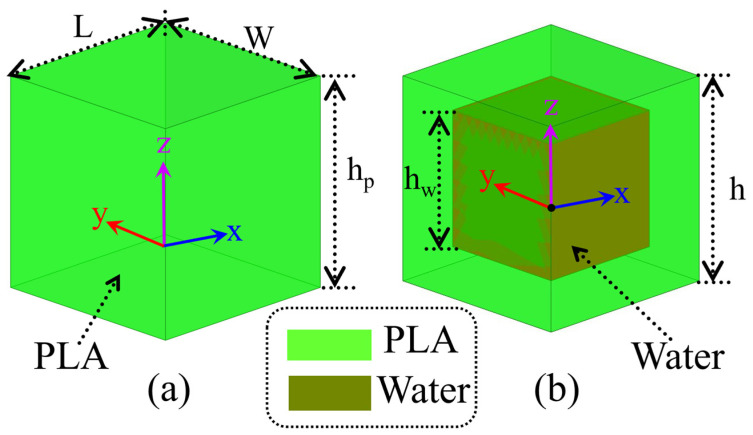
Different unit cells. (**a**) Dimetric view of the PLA-based unit cell; (**b**) Dimetric view of the proposed water-based unit cell.

The geometrical configuration of the proposed WBRTA unit cell is presented in Figure 1. Water and polylactic acid (PLA) dielectric materials are used to design the unit cells of the WBRTA. To avoid fabrication complexities and reduce production time and cost, metallic patches and vias are avoided in the proposed structure. Two different unit cell configurations are studied, namely PLA-based (Figure 1a) and water-based (Figure 1b). Due to its low relative permittivity (εᵣ ≈ 2.7), the PLA-based unit cell enables high transmission with good impedance matching to free space. In contrast, the water-based unit cell employs a dielectric medium with a much higher effective permittivity, resulting in stronger reflection due to the larger impedance mismatch with air. Although the static dielectric constant of water at room temperature is approximately 78, this value is only valid at microwave frequencies and is not applicable to millimeter-wave operation. At millimeter-wave frequencies, the dielectric response of water becomes strongly dispersive. Therefore, water is modeled using the predefined dispersive material from the CST Studio Suite library, which accurately represents its behavior in the millimeter-wave band. These characteristics are validated by the simulated transmission and reflection responses presented in Section 2.2.

The magnitude and phase of transmission and reflection can be controlled by varying the heights of PLA and water in the PLA- and water-based unit cells (i.e., h_P_ and h_w_), respectively. The proposed design achieves optimal transmission and reflection with unit cell dimensions of L = W = 7 mm and an overall height of h = 8 mm, selected for operation in the 28–32 GHz band, where L and W are subwavelength. The unit-cell height h is fixed for all configurations. After optimization, the unit-cell geometry is finalized and remains unchanged. The phase response is controlled by effective dielectric loading, with a fully solid PLA unit cell and a water-filled unit cell of height h_w_. The resulting 180° phase difference required for reflect–transmit operation stems from the strong dielectric contrast between PLA and water. The stable wideband transmission and reflection responses of the proposed unit cells ensure consistent phase control across the operating band, which is critical for reliable wideband beam steering in reflect–transmit array architectures. The electromagnetic (EM) performance of the PLA and water filled unit cells was evaluated using full wave simulations in CST Studio Suite. Each unit cell was analyzed under periodic boundary conditions to represent an infinite array and excited by a normally incident plane wave through Floquet ports.

### 2.2. Transmission and Reflection Characteristics

**Figure 2:**
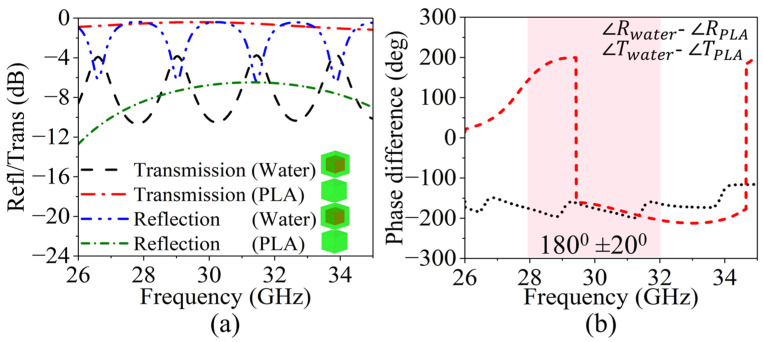
(**a**) Simulated reflection and transmission coefficients of the PLA-based and water-based unit cells. (**b**) Simulated phase responses of the unit cells in reflection and transmission modes (Note: The black curve represents the reflection phase difference between the PLA-based and water-based unit cells, while the red curve denotes the corresponding transmission phase difference).

The RTAs transmit half of the incident waves in the forward direction and reflect half of the incident waves in the backward direction; therefore, to develop a configuration suitable for a WBRTA, two unit cell configurations were simulated. To generate high reflection, the first unit cell encapsulates water with a thickness of h_w_ = 7 mm. As shown in Figure 2a, the water based unit cell provides high reflection around −0.5 dB over the frequency band from 28 to 32 GHz, except at two resonant frequencies located at 29 GHz and 31.5 GHz, where the reflection coefficient drops to −6.1 dB and −5.8 dB, respectively. At these frequencies, the transmission magnitude increases to −4.2 dB and −4.0 dB. These small deviations are caused by the dispersive dielectric behavior of water combined with the selected water height, h_w_ equals 7 mm, and do not affect the wideband reflect transmit functionality of the unit cell. These resonances may be attributed to the high dielectric constant and the height of the water. The transmission coefficient of these water-based unit cells reaches −4.2 dB at the resonant frequencies and remains lower than −6 dB across the rest of the operating band. Similarly, in the second unit cell configuration, the unit cell is a complete PLA structure with a thickness of hp = 8 mm. It is evident from Figure 2a that the PLA-based unit cell provides a transmission magnitude above −1.2 dB in the frequency band of 28 to 32 GHz, while its reflection coefficient remains around −6.5 dB over the same frequency range. These two unit cells provide an approximately 180° ± 20° phase difference in the frequency band of 28 to 32 GHz for both reflection and transmission, as shown in Figure 2b. The high reflection of the water-based unit cell, resulting from its very high permittivity, and the low reflection of the PLA-based unit cell, due to its low permittivity, combined with the 180° phase difference, enable beam focusing and beam tilting in the reflection mode. Similarly, the high transmission of the PLA-based unit cell and the low transmission of the water-based unit cell, together with the 180° phase difference, facilitate beam focusing and tilting in the transmission mode. By combining these two effects, the proposed WBRTA achieves beam splitting in the forward (transmission) and backward (reflection) directions, along with beam tilting in both modes. The stable 180° phase contrast maintained across the operating band enables consistent wavefront shaping, which is essential for accurate angular beam steering in reflect–transmit array systems.

## 3. WBRTA Design and Performance

### 3.1. WBRTA Array Configuration

A planar array of M × M reflecting or transmitting elements constitutes a reflectarray (RA) or transmitarray (TA) aperture. A source feed is placed on the *z*-axis at the point $z={\vec{r}}_{f}$. Two different unit cells (PLA-based and water-based) with distinct phase profiles are used as passive phase shifters to generate a beam in the desired direction by controlling the incident field produced by the source. According to array theory [23], the far-field radiation pattern for the transmission mode can be expressed as

(1) $$E\left(\theta ,\varphi \right)=\sum_{m=1}^{M}\sum_{n=1}^{M}{cos}^{qe}(\theta )\frac{{cos}^{qf}(\theta ){(\theta }_{f}(m,n))}{\left|{\vec{r}}_{m,n}-{\vec{r}}_{f}\right|}\cdot e^{-jk\left|{\vec{r}}_{m,n}-{\vec{r}}_{f}\right|)\left|X_{m,n}\right|e^{j\varphi_{m,n}}}$$

Here, *qf* and *qe* denote the feed pattern factor and the unit-cell pattern factor, respectively. Moreover, ${\vec{r}}_{m,n\;}$and $z={\vec{r}}_{f}$ represent the position vectors of the (*m*,*n*)th unit cell and the feed antenna, respectively. The parameter k is the free-space wavenumber, and *θf*(*m*,*n*) is the spherical angle in the feed’s coordinate system. The term $\left|X_{m,n}\right|$ corresponds to the reflection or transmission magnitude of the *mn*-th unit cell, obtained from the analysis of the PLA- and water-based unit cells. In specially fed transmit–reflect array antennas, a pencil beam can be generated in a desired direction û_0_(θ_0_, φ_0_) by assigning the phase of each unit cell (m, n) as

(2) $$\varphi_{m,n}=k(\left|{\vec{r}}_{m,n}-{\vec{r}}_{f}\right|-{\vec{r}}_{m,n}\cdot \hat{u})+\varphi_{c}$$

where $\hat{u}=\hat{x}sin$θcos*φ* + $\hat{y}sin$θsin*φ* + $\hat{z}cos$θ, and $\varphi_{c}\;$is a phase constant corresponding to an optimized value for a 1-bit RA/TA antenna. This formulation indicates that relative reflection and transmission phases are required, rather than absolute phases, when designing a reflect–transmit array.

**Figure 3:**
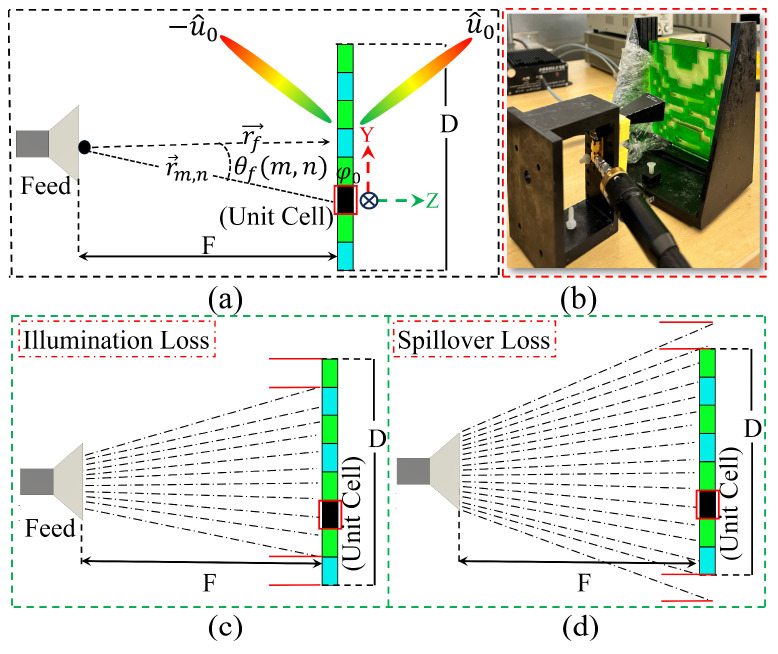
(**a**) Configuration of the antenna and the WBRTA array. (**b**) WBRTA mounted on the holder with the feed antenna positioned at the optimized focal distance of 72 mm. (**c**) Illustration of illumination loss in the WBRTA system. (**d**) Illustration of spillover loss in the WBRTA system.

It is evident from Equation (2) that the phase obtained from each unit cell varies with $\varphi_{c}$, leading to phase quantization errors if not properly optimized. Therefore, selecting an appropriate value of $\varphi_{c}\;$is essential for achieving optimal radiation performance in a 1-bit RTA. As demonstrated in Figure 3a–d, the focal distance between the proposed WBRTA and the source is optimized to F = 72 mm, corresponding to a −10 dB edge taper. This configuration ensures efficient aperture illumination and minimizes both illumination and spillover losses [24,25].

### 3.2. Simulated Results

**Figure 4:**
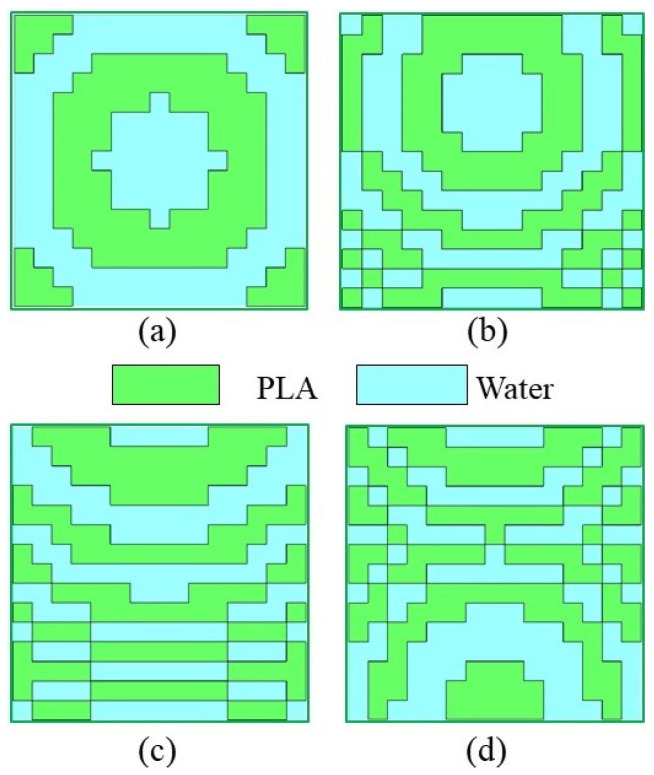
Configuration of the WBRTA array using water-based and PLA-based unit cells for beam tilting in different directions: (**a**) 0°, (**b**) 15°, (**c**) 30°, and (**d**) 45°.

**Figure 5:**
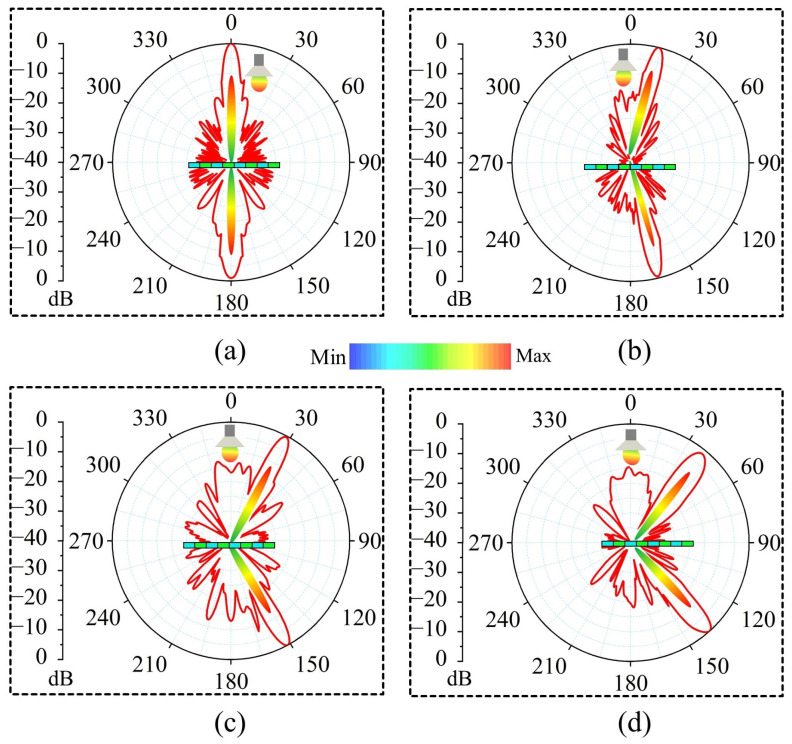
Simulated E-field radiation patterns of the WBRTA for beam tilting in different directions: (**a**) 0°, (**b**) 15°, (**c**) 30°, and (**d**) 45°.

To verify the beamforming and beam tilting functionalities of the WBRTA, simulations are performed in CST software 2024 using a time domain solver. The 15 × 15 elements array with a total aperture size of 105 × 105 mm^2^, and a WR-28 horn antenna feed with a peak gain of 15 dBi are considered. The proposed scenario can be seen from Figure 3a,b. The phase distribution for the beam-splitting operation at 0° in both reflection and transmission modes is initially obtained using a program developed in MATLAB software 2022, and the corresponding pattern is shown in Figure 4a. The design shows consistent radiation characteristics across the band, with small variations at the two resonance points due to slight changes in the unit cell magnitude and phase. It can be observed from Figure 5a that two pencil beams are formed in opposite directions at 30 GHz. The reflected beam is directed toward 0°, while the transmitted beam is aimed at 180°. Furthermore, SLLs of the proposed WBRTA are significantly lower compared to the main lobe. The SLL for the 0° WBRTA is 17.5 and 22 dB lower than the main beam in the reflection and transmission modes, respectively, and no grating lobes are observed.

**Figure 6:**
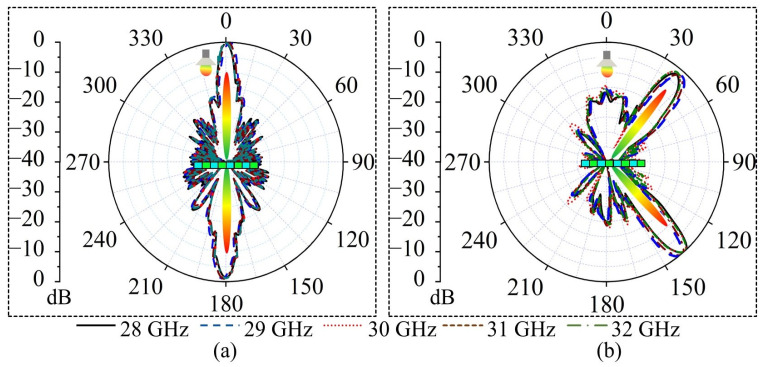
Simulated E-field radiation patterns of the WBRTA for beam tilting at different frequencies for (**a**) 0° and (**b**) 45°.

To form and tilt the beam at a 150° angle, the phase distribution is illustrated in Figure 4b. It is obvious from the radiation pattern shown in Figure 5b that when the incident wave hits the WBRTA, two pencil beams are generated, one reflects at 15° and the other gets transmitted in the 165° (180° − 15° = 165°) direction. For beam generation and beam tilting at 30° angle, the required phase distribution is portrayed in Figure 4c. The impinging wave splits into two, a half reflects to 30° and the other transmits with tilting toward 150° (180° − 30° = 150°) on interaction with the WBRTA. In the reflection, a 1° beam shift is observed. Finally, to tilt the beam at 45°, the phase distribution pattern on the WBRTA array displayed in Figure 4d is excited by the horn antenna. When normally incident wave hits the 45° WBRTA array, half of the wave reflects and half transmits. The beam focusing towards 45° in the reflection mode, and towards 135° (180° − 45° = 135°) in the transmission mode are depicted in Figure 5d. For this configuration, a 3° beam shift is observed in the reflection mode. It is worth noting that the performance of the array remains almost the same for the 0° and 45° configurations throughout the operating frequency band, as illustrated in Figure 6a,b.

## 4. Experimental Results

**Figure 7:**
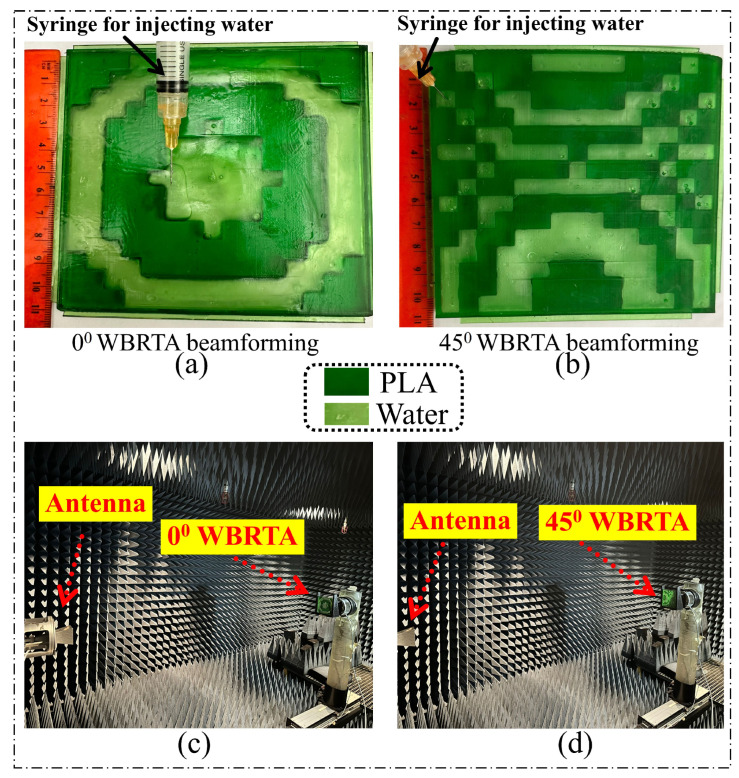
Water-based and PLA-based WBRTA array configurations for beam tilting: (**a**) configuration for 0°, (**b**) configuration for 45°, (**c**) radiation pattern measurement setup for the 0° WBRTA, and (**d**) radiation pattern measurement setup for the 45° WBRTA.

To practically validate the WBRTA array concept, the proposed designs for 0° and 45° beam tilting were fabricated through the ELEGOO SATURN–S 3D-printer (ELEGOO, Shenzhen, China) as displayed in Figure 7a,b, respectively. After fabrication, the prototypes were cured at a drying station. Water was subsequently injected into the water-based unit cells using a syringe. Water injection was performed using precision syringes with 0.5 mm gauge needles inserted through pre-designed filling ports located at the top surface of each unit cell. To eliminate air bubbles, the filling procedure employed a two-step approach: (1) initial vacuum degassing of distilled water for 10 min to remove dissolved gases, and (2) slow injection at a controlled rate of approximately 0.1 mL/min while tilting the array at 15° to allow trapped air to escape through the filling port. Moreover, to ensure long-term stability and operational reliability, several measures were implemented in the fabrication process. Each water-based unit cell was hermetically sealed using UV-curable epoxy resin applied to the injection ports immediately after filling, preventing water evaporation and leakage during operation. The epoxy curing process takes approximately 2–3 min under UV exposure, ensuring rapid sealing without water loss. Environmental stability tests conducted over a 30-day period showed negligible performance degradation (less than 0.3 dB gain variation), confirming the effectiveness of the sealing approach.

**Figure 8:**
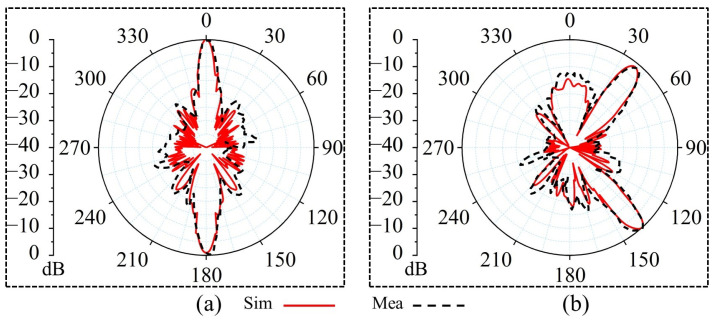
Simulated and measured co-polarized radiation patterns of the WBRTA: (**a**) 0° configuration and (**b**) 45° configuration.

**Figure 9:**
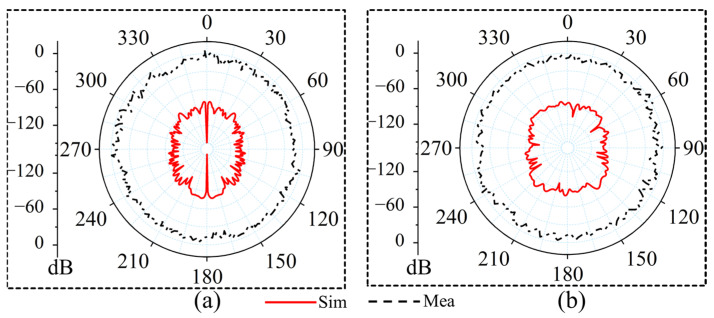
Simulated and measured normalized cross polarized radiation patterns of the (**a**) the 0° configuration and (**b**) the 45° configuration. In both cases, the measured cross polarization level is constrained by the cross polar discrimination of the transmit antenna, which sets the effective measurement floor and matches the observed values. The simulated cross polarized response is significantly lower than this limit, indicating a much higher intrinsic polarization purity of the proposed antenna that cannot be fully resolved in the measurement setup.

**Figure 10:**
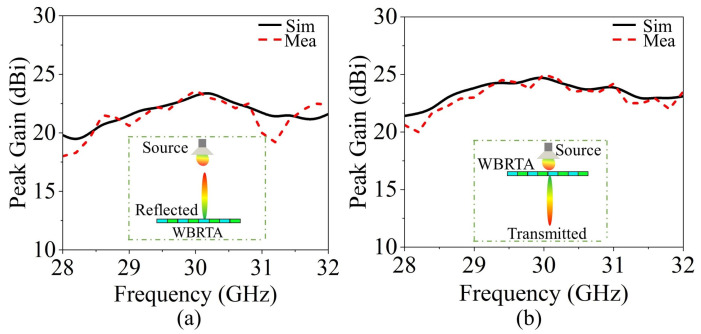
Simulated and measured peak gains. (**a**) In reflection mode. (**b**) In transmission mode.

The measurements of the 0° and 45° WBRTA arrays were performed using the setups shown in Figure 7c,d, respectively. The Ka-band horn antenna is positioned on the transmitting side, while the WBRTA array is located on the receiving side. The VNA is used to obtain the radiation measurement of both the 0° WBRTA and 45° WBRTA. As shown in Figure 8a,b, the measured copolarized radiation pattern (co-pol) of both WBRTA arrays validates the beam tilting and beam forming functionalities and is in good agreement with the simulated patterns. As the proposed WBRTAs do not use metal or vias; therefore, the mutual coupling between elements is very low [26,27], resulting in very low x-pol values (−81 dB) compared to those reported in the literature, as shown in Figure 9a,b. It is pertinent to mention that the measured cross-polarization performance is constrained by the test equipment rather than the WBRTA’s inherent characteristics. The WR-28 horn antenna used as the transmitter possesses a cross-polar discrimination specification of approximately −42 dB, which defines the system’s noise floor and coincides with the observed experimental values. Electromagnetic simulations indicate the WBRTA achieves cross-polarization levels reaching −80 dB, but this superior performance cannot be experimentally verified as it falls below the −42 dB measurement threshold. Consequently, while measurements validate that the cross-polarization is −42 dB or better, the computational analysis demonstrates that the antenna’s true polarization purity substantially exceeds what can be detected with the available instrumentation. The simulated and measured peak gains of the 0° WBRTA configuration are approximately 24 dBi for the reflected beam and 25.2 dBi for the transmitted beam, as shown in Figure 10a,b. The small variation in the simulated and measured patterns and gains could be attributed to fabrication imperfections and measurement setup errors. The comparison of the WBRTA with other recently reported RTAs in the literature is given in Table 1. It is clear from Table 1 that despite having a metalless structure and low SLL and x-pol, the proposed WBRTA provides a higher gain, a single-layer structure, and a low-cost fabrication than those in Table 1. It is evident that, despite its metalless single-layer structure and low sidelobe and cross-polarization levels, the proposed WBRTA achieves higher gain and significantly lower fabrication cost than existing designs reported in the literature. These characteristics confirm its suitability for wideband mmWave positioning and sensing platforms requiring high gain, angular selectivity, and robust wideband performance. Millimeter-wave positioning systems rely heavily on accurate angle-of-arrival (AoA) estimation, high angular resolution, and strong spatial selectivity. The proposed WBRTA directly supports these requirements through its low sidelobe levels, high polarization purity, wideband operation, and bidirectional beamforming capability. Specifically, the achieved sidelobe levels of −22 dB and −17 dB in the transmission and reflection modes, respectively, significantly suppress spurious radiation and reduce false angle detection, which is critical for reliable target localization. Moreover, the extremely low cross-polarization levels of the metalless design enhance polarization discrimination, thereby improving AoA estimation accuracy in multipath-rich environments. The wide operational bandwidth (28–32 GHz) further contributes to improved ranging resolution, which is essential for high-precision mmWave positioning. In addition, the bidirectional beamforming capability enables simultaneous illumination of multiple spatial regions, facilitating efficient coverage and spatial diversity for positioning-oriented sensing applications. Collectively, these characteristics make the proposed WBRTA a promising hardware platform for future mmWave positioning and sensing systems.

## 5. Conclusions

Single-layer and cost-effective WBRTA arrays have been introduced for millimeter-wave (mm-wave) applications. Unlike previously designed RTAs, the proposed RTAs do not use metal or vias; therefore, they offer a very simple and easy-to-fabricate structure. Despite their structural simplicity and cost-effectiveness, the proposed WBRTAs provide high gain, low sidelobe levels (SLLs), low x-pol, and beam steering of up to 45° in both the transmission and reflection modes simultaneously. Owing to their compatibility with low-cost 3D printing, the proposed WBRTAs offer significantly reduced fabrication costs compared to previously reported RTA designs. With these features, the proposed design represents a promising candidate for mm-wave applications. In addition, the demonstrated wideband bidirectional beamforming capability, low sidelobe levels, and high polarization purity make the proposed WBRTA a promising hardware platform for mmWave positioning-enabled wireless systems, where flexible beam control and angular coverage are key enablers. In addition to communication applications, the proposed WBRTA is well suited for positioning and sensing scenarios, where wide angular coverage, low sidelobe levels, and high polarization purity are required for reliable angle of arrival estimation and spatial detection. The demonstrated bidirectional beamforming capability allows simultaneous coverage of multiple regions, which is advantageous for future mmWave localization and tracking systems.

## Figures and Tables

**Table 1:** Comparison with previously published designs.

Ref.	Published Year	Peak Gain (dBi) TA/RA	TAE (%)	Height of TRA	SLL (dB)	Measured X-Pol (dB)	Metal-Less	Maximum Tilt Angle	Fabrication Cost
[17]	2024	23.6/23.1	13.3	12 mm	−12/−11	−26.9	No	±30°	Very High
[18]	2024	18/21	19	5 mm	−10/−11	−13.5	No	0°	High
[19]	2023	22.9/24.4	38.5	27 mm	−14/−11	−21.9	No	±50°	Very High
[20]	2025	11.3/16.6	16.61	5.2 mm	N/A	−28	No	0°	High
[21]	2023	25.7/24.1	17.9	12 m	−15.3	−22.5	No	±15°	High
[22]	2025	27.7	44.4	8 mm	−15/−12	−38	No	±0°	High
This work		25.2/24	47	8 mm	−22/−17	−42	Yes	±45°	Very Low

TAE = Total Aperture Efficiency; SLL = Sidelobe Levels; X-pol = Cross-polarization.

## Data Availability

The original contributions presented in this study are included in the article. Further inquiries can be directed to the corresponding author.
